# Pre-COVID-19 Physician Awareness of Mental Health Resources During and After Natural and Human-Made Disasters

**DOI:** 10.1017/dmp.2022.256

**Published:** 2022-11-03

**Authors:** Natasha Sood, Joshua P. Hazelton, Sue Boehmer, Robert P. Olympia

**Affiliations:** 1 Pennsylvania State College of Medicine, Hershey, PA, USA; 2 Division of Trauma, Acute Care and Critical Care Surgery, Pennsylvania State Health Milton S. Hershey Medical Center, Hershey, PA, USA; 3 Department of Public Health Sciences, Division of Biostatistics, Pennsylvania State College of Medicine, Hershey, PA, USA; 4 Department of Emergency Medicine and Pediatrics, Pennsylvania State Hershey Medical Center, Hershey, PA, USA

**Keywords:** climate change, frontline workers, health care workers, human-made disaster, mental health, natural disaster, physician mental health

## Abstract

**Objective:**

Physician mental health is critical during the recovery of natural and human-made disasters (NHDs), yet the accessibility of mental health resources to physicians has not been characterized. This study examined emergency medicine and trauma physician knowledge of and access to mental health resources in NHD settings.

**Methods:**

The survey was electronically disseminated to the American College of Emergency Physicians and the American Association of the Surgery of Trauma between February 4, 2020, and March 9, 2020. The 17-question survey assessed physician awareness and access to emergency preparedness resources at their institutions.

**Results:**

Of the responders, 86% (n = 229) were aware of written emergency response plans for their facility. While 31% were aware of the hospital’s mental health policies and resources outside of the emergency response plan, only 25% knew how to access these resources during and after NHDs. Finally, 10% reported the incorporation of mental health resources during institutional practice drills.

**Conclusions:**

Physicians reported knowledge of emergency preparedness policies; however, significant gaps remain in physician knowledge and access to mental health resources NHD settings. As NHDs increase on a global scale, it is critical for health systems to ensure accessible infrastructure to support the mental well-being of health professionals.

## Impact of Natural and Human-Made Disasters

The treatment of people affected by natural and/or human-made disasters (NHDs) is a critical aspect of emergency management, and physicians are amongst those at the front lines of administering this care.^1^ NHDs often necessitate that physicians work in areas of devastated health care infrastructure, profoundly limited resources, and surges in acute and chronic care patients.^2^ Thus, the mental health of hospital physicians is a key issue during and after NHDs.

Physicians are exposed to a triple threat as they are victims of the NHD, responsible for providing emergent and ongoing care for their community, and must deal with the complex decision making of staying to deliver care or moving to safe places to protect themselves and their families. A growing body of literature highlights that high stress environments can result in negative mental health outcomes and burnout in physicians, leading to decreased workplace productivity and decreased quality of patient care.^3–5^

Disasters such as the 2011 Fukushima Nuclear Disaster, 2012 Superstorm Sandy, 2015–2022 California wildfires, and the coronavirus disease (COVID-19) pandemic have underscored a critical gap in data on physician mental health following disasters.^6^ Prior to COVID-19, few studies have provided a systematic assessment of physician preparedness for a diverse set of health emergencies, much less the accessibility of mental health resources for physicians surrounding NHDs.^7^

The frequency and severity of global natural disasters have increased threefold since 1975, and the increased global burden of disease as a result of climate change and strained health care infrastructure threatens to compromise workforce mental health.^8,9^ Strong mental health support systems and system resilience will provide an environment in which physicians can provide essential medical care to affected populations during an NHD.

## Previous Research

Researchers at Harvard T. H. Chan School of Public Health developed The *Physician Emergency Preparedness Survey*, a 60-question survey that asks physicians about how they view preparedness for emergency situations.^8^ Specifically, this survey examined United States physicians’ assessments of their NHD preparedness, training, and perceived support needs. They found that approximately 44% hospital physicians did not know whether their institution had an emergency response plan.^8^ The study did not explore the accessibility of mental health resources to physicians in those settings.

## Objective

This study examined physicians’ knowledge of and access to mental health resources at their institutions surrounding NHDs. While this study was initiated 2 years before the COVID-19 pandemic took hold in the United States, the pandemic has further highlighted the importance of this work. A review of available studies in databases including PubMed and Google Scholar revealed no prior peer-reviewed national studies assessing physician awareness of mental health resources in the setting of NHDs.

## Methods

### Study Design and Sample

A 17-question survey collecting information on physician awareness of and access to emergency preparedness resources at their institutions was administered nationally to practicing emergency medicine and trauma surgeon physicians. The survey, which was approved by the American College of Emergency Physicians (ACEP) research team, was distributed electronically to members of ACEP and AAST from February 4, 2020, to March 9, 2020. No incentives were offered for participation. The Institutional Review Board (IRB) granted this study exempt status.

### Survey Design

Researchers at Penn State College of Medicine developed the survey based on the *Physician Emergency Preparedness Survey*.^8^ In 2019, the survey was initially validated by Pennsylvania State Health emergency medicine and trauma physicians, and subsequently approved by Emergency Medicine Practice Research Network (EMPRN) at ACEP.

The survey included 17 questions on details of institution-specific written emergency response plans and physician awareness of and access to mental health resources surrounding NHDs (Appendix A). Information on hospital demographic and setting, and type of medicine practiced was collected. Finally, the survey asked whether mental health resources and policies were discussed in disaster preparedness practice drills.

NHDs were defined as (1) natural disasters (eg, hurricanes, earthquakes, tornado, wildfires), (2) chemical, biological, radiological, nuclear, or explosives (CBRNE) incident, (3) mass outbreak, and (4) mass shooting. Mental health resources include, but are not limited to, a mental health professional available to physicians during and after NHD; trainings during and after NHD on how physicians are to deal with the traumatic consequences of the NHD; creation of “safe spaces” for physicians to process NHD consequences; mental health professional monitoring, limiting, and rotating individual physicians when providing care during and after NHD; and mental health screening of physicians during and after NHD.

### Analysis

Statistical analysis was performed using SPSS, version 25.0 (Statistical Package for the Social Sciences [SPSS] Inc., Chicago, IL, USA). Descriptive analysis was used to report frequencies for all items in the questionnaire for ACEP and AAST participants together. In addition, responses of physicians practicing at Level 1 Trauma Centers were compared to physicians practicing at Non-Level 1 using chi-square analysis to explore differences by the Trauma Center Level.

## Results

Responses were categorized by demographics, knowledge components of written emergency response plan, and physician views on mental health resources. There were no differences in responses by gender, but significant differences were found between Level 1 Trauma Center and Non-Level 1 Trauma Centers.

### Demographics

Of those who completed the survey (n = 229), 80% were white, 75% were male, and 80% were > 10 years out of graduate training; 76% reported working at a trauma center, and over 59% were based in urban inner-city environments (Table 1). Respondents practicing in Level 1 Trauma Centers were significantly more likely to be ≤ 10 years out of post-graduate training (*P* = 0.000) and to work in urban inner-city settings (*P* = 0.000).

**Table 1.** Demographics (responding *yes*)

Questionnaire item	Overall n (%)	Level 1 trauma center n (%)	Non-level 1 trauma center n (%)	95% CI of difference in proportion	*P*
n	229	114	115		
Attending	228 (99)	112 (98)	115 (100)	−0.046, 0.006	0.247
White ethnicity	183 (80)	88 (77)	94 (82)	−0.154, 0.054	0.394
> 10 Years post-graduate	179 (80)	79 (70)	99 (82)	−0.230, −0.010	0.000*
Trauma center (I, II, III, IV)	175 (76)	114 (100)	115 (100)	0.000, 0.000	
Male gender	172 (75)	82 (72)	90 (78)	−0.172, 0.052	0.268
Urban inner-city setting	136 (59)	91 (80)	45 (39)	0.295, 0.526	0.000*

*P < 0.05.

### Components of Written Emergency Response Plan

Of the respondents, 86% were aware of an emergency response plan for their facility, 72% knew the communication plan to link all providers and administrative staff at home or in the care setting, 70% knew the roles for each staff member, and 49% knew information sources for treating illnesses and injuries related to different kinds of emergencies and triage plans with the names of alternative locations of care. Only 20% of respondents indicated that this written response plan included policies that addressed physician mental health during and after an NHD (Table 2).

**Table 2.** Components of written emergency response plan (reporting *yes*)

Questionnaire item	Overall n (%)	Level 1 trauma center n (%)	Non-level 1 trauma center n (%)	95% CI of difference in proportion	*P*
*Components of written emergency response plan (reporting yes)*					
	229	114	115		
Aware of a written plan	196 (86)	105 (92)	91 (79)	0.040, 0.220	0.005*
Roles of staff members	161 (70)	85 (75)	76 (66)	−0.028, 0.208	0.161
Addresses diverse emergencies	111 (49)	61 (54)	50 (44)	−0.029, 0.229	0.129
Continuing operations plan	157 (69)	91 (80)	66 (57)	0.114, 0.347	0.000*
Communication plan to link providers and administrators in various settings	165 (72)	92 (81)	73 (64)	0.057, 0.284	0.004*
Triage plan and alternative locations of care	111 (49)	68 (60)	43 (37)	0.104, 0.356	0.001*
Patient communication protocols	27 (12)	17 (15)	10 (9)	−0.024, 0.144	0.145
Addresses physician mental health needs during and after an NHD	39 (20)	23 (22)	16 (18)	−0.064, 0.144	0.521
*Physician views of mental health resources (responding yes)*					
	229	114	115		
Aware of hospital NHD mental health policies	71 (31)	43 (38)	28 (24)	0.022, 0.2585	0.029*
How to access NHD hospital mental health policies	59 (26)	35 (31)	24 (21)	−0.013, 0.213	0.089
Aware of ACEP and AAST disaster guidelines	117 (51)	57 (50)	59 (52)	−0.150, 0.110	0.843

*P < 0.05*.*

On all items, physicians practicing at Level 1 Trauma Centers were more likely to be aware of the components of the written emergency response plan. However, there was no difference between physicians practicing at Level 1 and Non-Level 1 Trauma Centers in their knowledge of whether the written plan addressed mental health needs during and after an NHD (*P* = 0.521).

### Physician Views on Mental Health Resources

Outside of the written response plan, 31% of participants were aware of hospital mental health polices and policies during and after an NHD, and 26% knew how to access these mental health resources during and after an NHD. Finally, 10% reported accessing these resources during emergency preparedness practice drills (see Table 2).

Physicians working at Level 1 Trauma Centers were more likely to be aware of NHD mental health policies compared to those working at Non-Level 1 Trauma Centers (38% vs 24%; *P* = 0.029). However, there were no differences in their knowledge of means to access hospital NHD mental health resources, awareness of ACEP and AAST disaster guidelines, and use of these resources in practice drills.

## Discussion

This study provides a comprehensive assessment of physician knowledge of and access to mental health resources within institution-specific emergency response plans in the setting of NHDs prior to the COVID-19 pandemic. It also addresses physician views of emergency preparedness at their respective institution in multiple kinds of public health emergencies, including natural disasters, CBRNE, major outbreaks, or shootings. While physicians practicing at Level 1 Trauma Centers were more likely to be aware of the majority of components of their institution’s written emergency response plan, this did not hold true for awareness of the response plan addressing physician mental health needs surrounding an NHD. Of note, only 20% of all physicians were aware whether the response plan addressed physician mental health needs during and after an NHD. The results reveal critical gaps in emergency preparedness for physicians in that only 26% of Emergency Medicine and Trauma Surgery physicians know how to access mental health resources in the setting of an NHD, and 10% report actually using these resources during practice drills.

This study indicates that most institutions have written emergency response plans and physicians are aware of them.^10^ Yet, a significant portion of physicians are unaware of how to access the resources outlined in the preparedness protocols, especially as they pertain to mental health in the setting of NHDs.^8^ This suggests a lack of communication between emergency preparedness efforts and frontline workers.^8^ Finally, only 10% of physicians reported inclusion of mental health provisions during practice preparedness drills. Importantly, emergency preparedness drills are a fundamental tenet of emergency preparedness and a central pillar of Joint Commission accreditation, which most health centers seek.^8,11^

A comprehensive review of physician emergency preparedness by SteelFisher et al. (2015) demonstrated continuing barriers to physician preparedness, despite federal investment in physician and health system preparedness.^8^ This study emphasizes that emergency preparedness plans prior to the COVID-10 pandemic failed to adequately address communication about the existence of and accessibility of mental health resources during NHDs. The COVID-19 pandemic has further demonstrated that the level of preparedness for the psychological impact of NHDs on physicians has been grossly inadequate.^6,8,12^ As the COVID-19 pandemic progresses, 60% of physicians have reported experiencing burnout, up from the 54.4% burnout rate reported by Shanafelt et al. (2015).^4,5,13^ Yet, only 13% of physicians have sought treatment to address pandemic-related psychological concerns due to fear of stigma and harming their reputation.^13^

Additional examination of changes in the accessibility of mental health resources available to physicians in the setting of NHDs after the COVID-19 pandemic may reveal ways to improve emergency preparedness.^8^ The COVID-19 pandemic exemplifies the stress that frontline workers are under. Hospitals and health care system leaders must seek ways to implement and communicate accessible mental health resources to physicians.

Previous studies have reported adverse psychological reactions among frontline workers in the setting of the 9/11 terrorist attacks, wildfires, nuclear incidents, and major infectious disease outbreaks.^14^ However, no study has reported on the accessibility of mental health resources to frontline physicians surrounding NHDs. The COVID-19 pandemic underscores the need for training of public health emergencies, and the recovery provides a critical opportunity for the health systems to prepare their workforce for future crises. As large-scale public health crises like this cripple global supply chains, strain hospital resources, and limit hospital capacity, the increasing strain on personal health care becomes increasingly problematic.

### Strengths

This is the first study examining physician awareness and accessibility of mental health resources during NHDs in a national sample of Emergency Medicine and Trauma Surgery physicians who were members of ACEP and AAST. This study was conducted prior to the COVID-19 pandemic in the United States and provides a baseline that will allow the comparison of mental health resource accessibility in the post COVID-19 era. It will help assess whether the resources are now being better redirected toward this issue.

### Limitations

This study was conducted prior to the COVID-19 pandemic and may not be reflective of current physician attitudes and knowledge. Furthermore, this survey was administered to only Emergency Medicine and Trauma Surgery physicians as a sample of physicians working on the front lines during NHDs and should be expanded to all frontline workers during a diverse array of NHDs. Finally, this survey did not consider participants’ prior exposure to NHDs. However, this study provides a baseline to compare the impact of NHDs, like COVID-19, on physician mental health emergency preparedness. It reinforces the need for easily accessible mental health infrastructure in disaster preparedness protocols.

## Conclusion

With the increasing frequency and severity of NHDs in the setting of the climate crisis, the findings in this study provide much needed direction for the development of robust, easily accessible mental health resources for physicians on the front lines of emergency response. This study demonstrates that easily accessible mental health resources within emergency response plans are critical in the recovery from the COVID-19 pandemic and provides an opportunity for growth in recovery.

An understanding of the extent of mental stress levels among physicians in NHD will help formulate robust health policies to be adopted by hospitals and other agencies (ie, the government, state).^15^ This will improve resilience among health systems and physicians, thus improve their personal and professional lives and their patient interactions.

## Appendix A Survey

This survey will explore access to mental health resources in natural and/or human-made disaster (NHD) settings.

NHDs include, but are not limited to, a natural disaster (eg, hurricane, earthquake, tornado), chemical or biological incident, radiological or nuclear incident, major outbreak, mass shooting, or explosion.

Mental health resources include, but are not limited to, a mental health professional available to physicians during and after NHD; trainings during and after NHD on how physicians are to deal with the traumatic consequences of the NHD; creation of “safe spaces” for physicians to process NHD consequences; mental health professional monitoring, limiting, and rotating individual physicians when providing care during and after NHD; and mental health screenings of physicians during and after NHD.

### SECTION 1: DEMOGRAPHICS

1. Age: ___
2. State: *[drop down]*
3. Zipcode of Medical Center:
4. Ethnicity
  - Asian Indian or Alaska Native
  - Asian
  - Black or African American
  - Hispanic or Latino
  - Other
  - Two or more races
  - White
5. Gender
  - Female
  - Male
6. Indicate your professional status *(select one*):
  - Resident *(skip to question 8)*
  - Fellow *(skip to question 8)*
  - Attending
7. Years since graduate training
  - 0-5
  - 6-10
  - 11-15
  - 16-20
  - 21-25
  - 26-30
  - 31-35
  - 36-40
  - 41-45
  - 46+

### SECTION II: HOSPITAL SETTING

1. Select the level trauma center of your institution
  - Level I
  - Level II
  - Level III
  - Level IV
  - Not a trauma center
  - Not sure
2. In what setting is your institution located?
  - Inner city
  - Urban
  - Suburban
  - Rural
  - Not sure
3. Please select all that apply to the capacity in which you provide most of your patient care. *[multiple answers]*
  - Emergency Department
  - Trauma Surgery
  - A hospital setting
  - A setting that is not part of a hospital
  - Pediatric (only)
  - Adult (only)
  - Pediatric and adult

### SECTION III: EMERGENCY RESPONSE PLAN

1. In this department, is there a written emergency response plan?
  - Yes, there is a written plan.
  - No, there is not a written plan. (*Skip to question 14.)*
  - Don’t know. (*Skip to question 14.)*
2. Does this written plan include each of the following? (*Responses: Yes, No, Don’t Know*)
  - A description of roles for each staff member
  - Information sources for treating illnesses and injuries related to different kinds of emergencies
  - A continuing operation plan for treating routine and overflow patients
  - A communication plan to link all providers and administrative staff at home or in the care setting
  - A plan to reach out to your current patients—for example, by updating the practice website or reaching high-risk patients through phone messages
  - A patient triage plan with the names of the alternative locations of care
3. Does this written plan include policies that address physician mental health needs during and after an NHD?
  - Addresses needs both during and after
  - Addresses needs only during NHD
  - Addresses needs only after NHD
  - Does not address needs during or after NHD.
  - Don’t know
4. Are you aware of your hospital mental health polices and resources during and after an NHD?
  - Yes
  - No (*Skip to question 16.)*
5. Do you know how to access these hospital mental health resources during and after an NHD?
  - Yes
  - No
6. Are you aware of and familiar with the national ACEP and AAST disaster preparedness guidelines?
  - Both
  - Only ACEP
  - Only AAST
  - Neither
7. In past practice drills in which this emergency plan was used, were any mental health resources designed for physicians made available to you during these drills?
  - Yes
  - No
  - Don’t know

*Mental health resources include, but are not limited to, a mental health professional available to physicians during and after NHD; trainings during and after NHD on how physicians are to deal with the traumatic consequences of the NHD; creation of “safe spaces” for physicians to process NHD consequences; mental health professional monitoring, limiting, and rotating individual physicians when providing care during and after NHD; and mental healths screening of physicians during and after NHD.*
